# No clear associations between subjective memory concerns and subsequent change in cognitive function: the PATH through life study

**DOI:** 10.1007/s10433-022-00694-2

**Published:** 2022-03-28

**Authors:** Ying Xu, Jane Warwick, Ranmalee Eramudugolla, Hamidul Huque, Kaarin J. Anstey, Ruth Peters

**Affiliations:** 1 Neuroscience Research Australia (NeuRA), Margarete Ainsworth Building, Barker Street, Randwick, NSW 2031 Australia; 2 School of Psychology, University of New South Wales, Sydney, NSW 2052 Australia; 3 Warwick Clinical Trials Unit, Warwick Medical School, University of Warwick, Gibbet Hill Campus, Coventry, CV4 7AL UK; 4 School of Public Health, Imperial College London, London, W2 1PG UK

**Keywords:** Older adults, Dementia, Executive, Attention, Processing speed, Population-based

## Abstract

The literature on subjective memory concerns (SMC) as a predictor for future cognitive decline is varied. Furthermore, recent research has pointed to additional complexity arising from variability in the experience of SMC themselves (i.e. whether they are remitting or sustained over time). We investigated the associations between SMC and objectively measured cognition in an Australian population-based cohort. Four waves (4-year intervals between waves) of data from 1236 participants (aged 62.4 ± 1.5 years, 53% male) were used. We categorized participants as experiencing SMC, when they indicated that their memory problems might interfere with their day-to-day life and/or they had seen a doctor about their memory. SMC was categorized as “no” reported SMC, “remitting”, “new-onset” or “sustained” SMC. Cognitive assessment of immediate and delayed recall, working memory, psychomotor speed, attention and processing speed were assessed using a neuropsychological battery. Eighteen percent of participants were characterised as having SMC: 6% (77) “remitting”, 6% (77) “new-onset” and 6% (69) “sustained” SMC. There was no consistent evidence for an association between SMC and subsequent decline in cognition. However, SMC was associated with poorer performance on contemporaneous tasks of attention and processing speed compared to “no” SMC. Asking about SMC may indicate a current decline in cognitive function but, in this sample at least, did not indicate an increased risk of future decline.

**Supplementary Information:**

The online version contains supplementary material available at 10.1007/s10433-022-00694-2.

## Introduction

In recent years, there has been substantial interest in the potential for subjective perception of cognitive decline, particularly, subjective concerns about memory, as a prognostic indicator for increased risk of Mild Cognitive Impairment (MCI) or dementia, see recent reviews (Wang et al. 2020; Wion et al. 2021). This follows on from the 2014 publication of a conceptual framework for research in subjective cognitive decline (SCD) in Alzheimer’s Disease (AD) (Jessen et al. 2014), and it is supported by imaging outputs that have highlighted a potential preferential vulnerability of AD relevant regions in SCD (Wang et al. 2020), including associations between hippocampal atrophy and subjective memory decline specifically (Cherbuin et al. 2015).

However, despite the growth of the literature in this area, estimates of prevalence and progression rates (conversion to MCI or dementia) are inconsistent. This lack of consistency may be driven in part by different sample characteristics, (e.g. a recent study reported sex differences (Jones et al. 2019)), or by variation in the experience of subjective decline itself as a remitting or stable condition (Si et al. 2020). For example, sustained SCD has been associated with a doubling in the risk of developing AD, a finding not seen for non-sustained SCD (Wolfsgruber et al. 2016). A greater understanding of the relationship between subjective and objective cognitive decline over time is needed before we can understand whether, how, and in whom we can classify subjective impairment as a reliable indicator for objective decline.

Our aim was to examine the relationship between stable and remitting subjective memory concerns (SMC) and contemporaneous and subsequent objective cognitive decline. We hypothesized that stable SMC would show the strongest association with objective measures of decline.

## Participants and methods

### Participants and procedures

The Personality and Total Health (PATH) through life study is a longitudinal population study with participant reassessment visits approximately every 4 years from baseline, the full design of which has been described elsewhere (Anstey et al. 2020, 2012). Briefly, participants who were residents of the cities of Canberra and Queanbeyan, Australia, were recruited randomly from the electoral roll (voting is compulsory in Australia). The current investigation focused on the oldest of three cohorts in the study, those aged 60–64 at baseline, and the first four waves of data collection, from 2001–2002 (wave 1) (*n* = 2551), wave 2 (2005 to 2006, *n* = 2222), wave 3 (2009 to 2010, *n* = 1973), and wave 4 (2013 to 2015, *n* = 1645). At each wave of data collection, participants completed a face-to-face survey collecting data on demographic characteristics, mental and physical health and lifestyle. A few weeks after the survey, participants completed standardised assessments of cognitive and physiological functions, e.g. blood pressure, administered by a trained interviewer. The study was approved by the Australian National University Human Research Ethics Committee. Written informed consent was obtained from all participants.

### Assessment of subjective memory concerns (SMC)

SMC was assessed at wave 1 and 2 using three questions (1) “Do you feel you can remember things as well as you used to? That is, is your memory the same as it was earlier in life?”. Participants who responded “no” or “depends/sometimes” were also asked (2) “Does this memory problem interfere in any way with your day to day life?” (“no”, “yes”, “don`t know”), and (3) “Have you seen a doctor about your memory?” (“no”, “yes”). We categorized participants as experiencing SMC, when they indicated that their memory problem might interfere with their day to day life (answers of “yes” or “don`t know” to question 2), and/or that they had seen a doctor about their memory (“yes” to question 3). Participants were categorized as not experiencing SMC, when they responded “yes” to the first question, or responded “no” or “depends/sometimes” to the first question but “no” to questions 2 and 3.

SMC was further categorized as “sustained” (i.e. reported at waves 1 and 2, approximately 4 years apart), “remitting” (SMC reported only at wave 1), “new-onset” (reported only at wave 2) and “no” SMC (neither wave).

### Assessment of objective cognitive function

Assessment of cognition was via a neuropsychological battery. This included, the California Verbal Learning Test (CVLT) (memory, immediate and delayed recall), modified for an epidemiological context (Anstey et al. 2012; Delis 1987). The Symbol Digit Modalities Test (SDMT (Smith 1982)) and trail making test part A and B (Reitan 1985) (processing speed, attention), the Digit Span Backwards (Wechsler 1945) (working memory) and the Purdue pegboard (dominant, non-dominant and both hand(s)) (Tiffin 1968) (psychomotor speed). Cognitive test scores from waves 2–4 were used in the current analyses, with the exception of the CVLT delayed recall, for which only wave 2 and 3 data were used, as wave 4 data were unavailable. Time (seconds) used for trail making tests part A and B was log-transformed to form normal distributions, and the coefficients were exponentiated. Cognitive test scores other than trail making tests part A and B were z transformed (i.e. mean scores at wave 2 of 0 and standard deviation (SD) of 1), with higher scores indicating better performance. The Mini-Mental State Exam (MMSE) was used as a global measure of cognition (Folstein et al. 1975).

### Other measures

Baseline covariates relevant to cognition and SMC included Body Mass Index (BMI, kg/m^2^) calculated using self-reported weight and height, hypertension (defined as a systolic blood pressure of > 140 mmHg and/or diastolic blood pressure of > 90 mmHg, on or off antihypertensive medication), depression and anxiety and physical activity (Anstey et al. 2019). Depression and anxiety were assessed using the self-administered Goldberg Anxiety (9-item) and Depression scale (9-item) (Goldberg et al. 1988). Participants with greater than five answers of “yes” (versus “no”) on the anxiety scale were classified as having anxiety symptoms, while participants with more than two answers of “yes” (versus “no”) on the depression scale were classified as having depression symptoms. Self-reported physical activity was categorised by vigour (mild, moderate and vigorous) and frequency (Australian Institute of Health and Welfare 2000; Northey et al. 2019).

### Statistical analyses

A linear mixed model was used. SMC category (“remitting” coded as “1”, “new-onset” as “2”, “sustained” as “3” and “no” SMC as “0”) was the exposure variable of interest. Cognitive change between waves 2–4 were the dependent variables, i.e. the measures of change in cognitive performance that were used in the analyses were subsequent to the assessment of self-reported SMC. Time was coded as 0 for wave 2, 1 for wave 3 and 2 for wave 4. The model was adjusted for baseline covariates measured at wave 1, including symptoms of anxiety and/or depression, BMI, hypertension, and self-reported following items: age, sex, English speaking, total years of education, diabetes, traumatic brain injury, smoking and physical activity. Continuous variables, age, BMI and total years of education were centred at their mean values. Other categorical variables were coded as absent/ “0″ versus present/”1″. Correlation between covariates were checked and not identified (correlation coefficient all < 0.4). Random effect of the intercept was added in all models. Effect modification of sex was checked, and analyses were rerun by sex when effect modification was identified.

Participants were excluded from analyses if they missed any SMC measure(s) at waves 1 and 2, missed cognitive tests, or had unavailable data for covariates in the models. To adjust for bias caused by attrition, we ran sensitivity analyses using inverse probability weighting (Lawrence Rasouliyan 2016). The probability of being included in the analyses was calculated based on general cognition at baseline (MMSE score) and the covariates that were adjusted for in the linear mixed model. Additional sensitivity analyses were conducted by repeating the analyses in those who did not have any anxiety or depression symptoms at baseline (assessed on the Goldberg Anxiety and Depression scale). All analyses were performed using SAS statistical software version 9.4.

## Results

Figure S1 shows how the analytic sample was achieved. There were 1236 participants (aged 62.4 ± 1.5 years, 53% male) included in the analytic sample with a mean age at baseline of 62.4 (Standard Deviation (SD) 1.5), 53% of whom were male (Table S1). This represented 75% of the 1645 participants who were followed up at wave 4. Baseline characteristics of those with “no”, “remitting”, “new-onset” and “sustained” SMC are presented in Table 1. Of the 1236 analysed participants, 1013 (82%) were categorized as having “no”, 77 (6%) “remitting” (SMC reported only at wave 1), 77 (6%) “new-onset” (reported only at wave 2), and 69 (6%) “sustained” SMC (at both waves). Baseline characteristics of participants who were included in the current analyses and those who were lost to follow-up or excluded are shown in Table S1.

**Table 1:** Characteristics by subjective memory concerns at wave 1 and 2

Characteristics	Subjective memory concerns wave 1 and 2			
	No	Remitting	New-onset	Sustained
	*n* = 1013	*n* = 77	*n* = 77	*n* = 69
*Demographic at wave 1*				
Female, *n* (%)	487 (48.1)	36 (46.8)	33 (42.9)	24 (34.8)
Age, years, mean ± SD	62.5 ± 1.5	62.4 ± 1.4	62.3 ± 1.6	62.5 ± 1.5
Education, years, mean ± SD	14.2 ± 2.5	14.7 ± 2.7	14.4 ± 2.8	14.4 ± 3.1
Caucasian (versus Asian and other), *n* (%)	987 (97.4)	73 (94.8)	77 (100)	67 (97.1)
English-speaking, *n* (%)	927 (91.5)	69 (89.6)	72 (93.5)	63 (91.3)
*Dementia risk factors at wave 1*				
Body mass index, kilogram/meters^2^, median (IQR)	26 (23.8, 28.7)	27.3 (24, 30.1)	26.3 (23.8, 29.1)	26.5 (24.1, 29.1)
Diabetes (self-reported), *n* (%)	62 (6.1)	3 (3.9)	7 (9.1)	3 (4.4)
Hypertension (SBP > 140 mmHg or DBP > 90 mmHg, on or off antihypertensives), *n* (%)	509 (50.3)	43 (55.8)	47 (61)	29 (42)
SBP, mmHg, mean ± SD	139.6 ± 19	139.5 ± 17.4	141.4 ± 18.8	131.9 ± 18.9
DBP, mmHg, mean ± SD	82.9 ± 10.3	83.8 ± 9.9	84.7 ± 10.9	80.8 ± 10.5
Traumatic brain injury (self-reported), *n* (%)	43 (4.2)	6 (7.8)	3 (3.9)	3 (4.4)
Score > 2, Goldberg depression scale, *n* (%)	150 (14.8)	33 (42.9)	29 (37.7)	32 (46.4)
Score > 5, Goldberg anxiety scale, *n* (%)	72 (7.1)	15 (19.5)	10 (13)	17 (24.6)
Smoking (past or current versus never), *n* (%)	446 (44)	31 (40.3)	30 (39)	31 (44.9)
*Physical activities*				
Mild, less frequent than “three times a week or more”, *n* (%)	261 (25.8)	28 (36.4)	27 (35.1)	21 (30.4)
Moderate, less frequent than “once or twice a week”, *n* (%)	273 (27)	31 (40.3)	23 (29.9)	32 (46.4)
Vigorous, less frequent than “one to three times a month”, *n* (%)	556 (54.9)	56 (72.7)	43 (55.8)	49 (71)
Cognitive performance at wave 2 or changes between waves*				
Mini-Mental State Examination, median (IQR)	30 (29, 30)	30 (29, 30)	30 (29, 30)	30 (29, 30)
Change in scores from wave 2 to 3	0 (− 1, 0)	0 (− 1, 0)	0 (− 1, 0)	0 (− 1, 0)
Change in scores from wave 3 to 4	0 (− 1, 0)	0 (− 1, 0)	0 (− 1, 1)	0 (− 1, 0)
California Verbal Learning Test immediate recall, mean ± SD	7.3 ± 2.2	6.8 ± 2.2	6.6 ± 2.1	6.9 ± 2.1
Change in scores from wave 2 to 3	− 0.4 ± 2	− 0.4 ± 2.2	0 ± 2.3	− 0.7 ± 1.9
Change in scores from wave 3 to 4	− 1.5 ± 2.1	− 1.3 ± 1.9	− 1.4 ± 2.1	− 1.2 ± 2.3
California Verbal Learning Test delayed recall, mean ± SD	6.4 ± 2.3	6.1 ± 2.2	6 ± 2.3	6 ± 2.3
Change in scores from wave 2 to 3	− 0.3 ± 2.1	− 0.4 ± 1.8	0 ± 2	− 0.7 ± 1.9
Symbol Digit Modalities, mean ± SD	51.2 ± 8.9	50.1 ± 9.1	50.4 ± 8.2	48.5 ± 7.4
Change in scores from wave 2 to 3	− 1.9 ± 5.8	− 2.5 ± 5.2	− 1.6 ± 4.8	− 1.6 ± 6
Change in scores from wave 3 to 4	− 2.4 ± 6.3	− 3.2 ± 6.6	− 2.4 ± 5.4	− 1.9 ± 5.6
Trail making test part A time, seconds, median (IQR)	32 (26, 38)	36 (28, 44)	33 (28, 40)	32 (27, 40)
Change in time from wave 2 to 3	2 (− 4, 8)	0 (− 7, 7)	1 (− 5, 7)	0 (− 4, 7)
Change in time from wave 3 to 4	1 (− 4, 7)	3 (− 3, 10)	0 (− 4, 6)	2 (− 2, 9)
Trail making test part B time, seconds, median (IQR)	70 (58, 88)	76 (58, 89)	74 (60, 88)	75 (58, 87)
Change in time from wave 2 to 3	5 (− 7, 17)	3 (− 6, 13)	4 (− 6, 18)	6 (− 8, 20)
Change in time from wave 3 to 4	6 (− 7, 20)	9 (− 6, 34)	8 (− 12, 23)	8 (− 8, 33)
Digit span backwards, mean ± SD	5.4 ± 2.2	5.8 ± 2.3	5.2 ± 2	5 ± 2.3
Change in scores from wave 2 to 3	− 0.2 ± 1.8	0 ± 1.7	0.1 ± 1.9	− 0.1 ± 1.8
Change in scores from wave 3 to 4	0 ± 1.8	− 0.5 ± 2.1	0 ± 1.9	− 0.1 ± 1.9
Purdue pegboard dominant hand, mean ± SD	13.7 ± 1.9	13.3 ± 2.2	13.3 ± 2.2	13.4 ± 1.7
Change in scores from wave 2 to 3	− 1.2 ± 1.9	− 1.2 ± 1.8	− 1.2 ± 2	− 1.6 ± 1.8
Change in scores from wave 3 to 4	− 0.6 ± 2.1	− 0.5 ± 2	− 0.8 ± 1.7	− 0.3 ± 1.9
Purdue pegboard non-dominant hand, mean ± SD	12.9 ± 1.8	12.4 ± 1.8	12.9 ± 2.1	12.1 ± 1.8
Change in scores from wave 2 to 3	− 1.1 ± 1.9	− 1.1 ± 2.1	− 1.4 ± 1.7	− 0.7 ± 1.8
Change in scores from wave 3 to 4	− 0.5 ± 2.1	− 0.5 ± 1.7	− 0.7 ± 1.9	− 0.7 ± 2.2
Purdue pegboard both hands, mean ± SD	10.6 ± 1.7	10.3 ± 1.8	10.3 ± 2.2	10.2 ± 1.7
Change in scores from wave 2 to 3	− 1 ± 1.6	− 0.8 ± 1.6	− 1.1 ± 1.6	− 0.7 ± 1.7
Change in scores from wave 3 to 4	− 0.4 ± 1.9	− 0.8 ± 1.7	− 0.3 ± 1.6	− 0.9 ± 1.6

*DBP* denotes diastolic blood pressure, *SBP* systolic blood pressure, *SD* standard deviation, *IQR* interquartile range

*For trail making test part A and B time, a positive number indicates more time used in later wave than earlier wave, whereas a negative number indicates less time used in later wave than earlier wave. For all the other cognitive performance tests, a positive number indicates a higher score in later wave than earlier wave, whereas a negative number indicates lower score in later wave than earlier wave

### *SMC and contemporaneous cognitive performance at wave 2 (Table **2**, column a)*

**Table 2:** Associations between subjective memory concerns and cognition in the linear mixed model^a^

	Differences in			
	Cognitive performance at wave 2 (column a)		Cognitive change between waves over the subsequent 8 years (column b)	
	β_2_^b^ (95% CI)	P value	β_3_^b^ (95% CI)	P value
CVLT immediate recall		0.02*		0.24
“Remitting” vs “no”	−0.23 (−0.43, −0.02)*	0.03*	0.03 (−0.08, 0.15)	0.59
“New-onset” vs “no”	−0.25 (−0.45, −0.04)*	0.02*	0.12 (0, 0.23)*	0.05*
“Sustained” vs “no”	−0.12 (−0.33, 0.1)	0.29	−0.01 (−0.13, 0.11)	0.90
CVLT delayed recall		0.36		0.20
“Remitting” vs “no”	−0.14 (−0.36, 0.08)	0.23	−0.04 (−0.24, 0.17)	0.72
“New-onset” vs “no”	−0.15 (−0.37, 0.07)	0.19	0.11 (−0.09, 0.32)	0.28
“Sustained” vs “no”	−0.09 (−0.32, 0.15)	0.47	−0.19 (−0.41, 0.02)	0.08
Symbol Digit Modalities		0.10		0.15
“Remitting” vs “no”	−0.13 (−0.36, 0.09)	0.24	−0.08 (−0.16, 0)	0.06
“New-onset” vs “no”	−0.07 (−0.29, 0.16)	0.56	0.02 (−0.06, 0.1)	0.68
“Sustained” vs “no”	−0.28 (−0.51, −0.04)*	0.02*	0.05 (−0.04, 0.14)	0.27
Trails A		0.04*		0.27
“Remitting” vs “no”	1.08 (1.01, 1.15)*	0.02*	0.99 (0.96, 1.02)	0.57
“New-onset” vs “no”	1.06 (1, 1.14)	0.06	0.97 (0.94, 1)	0.08
“Sustained” vs “no”	1 (0.93, 1.07)	0.97	1.01 (0.98, 1.05)	0.51
Trails B		0.73		0.94
“Remitting” vs “no”	1.01 (0.93, 1.09)	0.81	1.01 (0.97, 1.04)	0.79
“New-onset” vs “no”	1.03 (0.95, 1.11)	0.47	1 (0.97, 1.04)	0.82
“Sustained” vs “no”	1.04 (0.96, 1.13)	0.35	0.99 (0.95, 1.03)	0.61
Digit span backwards		0.05*		0.36
“Remitting” vs “no”	0.2 (−0.02, 0.41)	0.07	−0.04 (−0.14, 0.05)	0.37
“New-onset” vs “no”	−0.12 (−0.33, 0.1)	0.29	0.07 (−0.02, 0.17)	0.14
“Sustained” vs “no”	−0.19 (−0.42, 0.04)	0.10	0.01 (−0.1, 0.11)	0.91
Purdue pegboard (dominant)		0.31		0.70
“Remitting” vs “no”	−0.17 (−0.39, 0.05)	0.13	0.03 (−0.09, 0.15)	0.58
“New-onset” vs “no”	−0.11 (−0.33, 0.11)	0.31	−0.06 (−0.18, 0.06)	0.33
“Sustained” vs “no”	−0.11 (−0.34, 0.13)	0.37	−0.02 (−0.14, 0.11)	0.76
Purdue pegboard (non-dominant)		0.01*		0.15
“Remitting” vs “no”	−0.26 (−0.49, −0.03)*	0.03*	−0.02 (−0.15, 0.11)	0.74
“New-onset” vs “no”	0.01 (−0.22, 0.24)	0.92	−0.15 (−0.27, −0.02)*	0.03*
“Sustained” vs “no”	−0.34 (−0.58, −0.09)*	0.007*	0.03 (−0.11, 0.16)	0.67
Purdue pegboard (both)		0.56		0.68
“Remitting” vs “no”	−0.1 (−0.33, 0.13)	0.39	−0.05 (−0.17, 0.07)	0.45
“New-onset” vs “no”	−0.13 (−0.36, 0.1)	0.27	0.03 (−0.09, 0.15)	0.60
“Sustained” vs “no”	−0.08 (−0.32, 0.16)	0.52	−0.05 (−0.17, 0.08)	0.44

*CVLT* denotes California Verbal Learning Test, *CI* confidence interval

^a^CVLT immediate recall, Symbol Digit Modalities, Trails A and B, digit span backwards and Purdue pegboard wave 2 to wave 4; CVLT delayed recall wave 2 to wave 3. For CVLT delayed recall, it is the subsequent 4 years, rather than 8 years

^b^Cognitive test scores = intercept + β_1_ X time (i.e. wave 2 coded as 0, wave 3 coded as 1 and wave 4 coded as 2) + β_2_ X subjective memory concerns category (0 = ”no”, 1 = ”remitting”, 2 = ”new-onset”, 3 = ”sustained”) + β_3_ X time X subjective memory concerns category + adjusted variables

Adjusted for age, sex, English speaking, education, symptoms of anxiety and/or depression, body mass index, self-reported diabetes, hypertension, traumatic brain injury, smoking and physical activity

Time (seconds) used for trail making tests part A and B was log-transformed to form normal distributions. For ease of interpretation, we exponentiated the coefficients for trail making tests part A and B, e.g. an exponentiated coefficient of 1.01 represents a 1% increase in time

Linear mixed models showed some relationships between categories of SMC and contemporaneous cognitive performance on immediate recall, processing and psychomotor speed.

Specifically, for CVLT immediate recall, compared to participants who had “no” SMC, those who had “remitting” and “new-onset” SMC demonstrated poorer performance, with effect sizes of −0.23 (95% Confidence Interval (CI) −0.43 to −0.02, *p* = 0.03) for “remitting” SMC, and −0.25 (95% CI −0.45 to −0.04, *p* = 0.02) for “new-onset” SMC, respectively. For Purdue pegboard performance using the non-dominant hand those who had “remitting” and “sustained” SMC showed poorer performance with effect sizes of −0.26 (95% CI −0.49 to −0.03, *p* = 0.03) and −0.34 (95% CI −0.58 to −0.09, *p* = 0.007), respectively. Finally, those with “remitting” SMC also used 8% more time to complete the trail making test part A compared to those with no SMC (95% CI 1% to 15%, *p* = 0.02). “Sustained” SMC was associated with worse SDMT when compared to those with “no” SMC, with effect size of -0.28 (95% CI −0.51 to −0.04, *p* = 0.02).

### *SMC and subsequent decline in cognitive function between waves 2–4 (8 years, Table **2** column b)*

There were no relationships between “sustained” and “remitting” SMC and subsequent cognitive decline in any domain. Participants with “new-onset” SMC showed mixed outcomes. Specifically, those with “new-onset” SMC had 0.15 SD steeper decline between waves in Purdue pegboard performance using the non-dominant hand (95% CI −0.27 to −0.02, *p* = 0.03) but a 0.12 SD lesser decline in immediate recall performance relative to “no” SMC (95% CI 0 to 0.23, *p* = 0.05). No other difference in the speed of decline between waves during the subsequent 8 years was observed. Estimations for the covariates can be found in Table S2.

### Sex difference and sensitivity analyses

There was no effect modification of sex in the contemporaneous associations between SMC and objectively measured cognitive function. The association of SMC and subsequent decline differed between males and females only for immediate recall (*p* = 0.01). There were no significant results when the analyses were rerun by sex although point estimates suggested a lessor decline in women than in men for both “sustained” and “remitting” SMC compared to “no” SMC. Inverse probability weighting and repeated analyses conducted in those who did not have any anxiety or depression symptoms at baseline found similar results (Table S3).

## Discussion

In this population study of older adults in the Canberra region of Australia, around a third of reported SMC could be classified as “sustained”, that is, present at baseline and wave 2 assessment four years later. Compared to those with “no” SMC, neither presence of self-reported “remitting” nor “sustained” SMC was associated subsequent decline in cognition. However, there were some associations seen for contemporaneous assessment of cognition. Overall, our hypothesis was only partially supported with “sustained” SMC showing the strongest associations with poorer contemporaneous performance on the SDMT and Purdue pegboard (non-dominant hand) performance but without similar findings for the other neuropsychological tests. Our results imply that participants were self-identifying some level of contemporaneous cognitive impairment but that this did not predict subsequent decline. Although the evidence base in this area is mixed our results are not inconsistent with prior studies, for example the proportion of our study population (11.8% at wave 1 and 11.8% at wave 2) who reported SMC, is comparable to the 12.6% seen in a 2007 study of 2389 participants in Germany aged 75 to 89 years, where SMC was assessed by a question, “Do you feel like your memory is becoming worse?”, with answers of “yes, this worries me” (versus “yes, but this does not worry me” or “no”) (Jessen et al. 2007), and congruent with a European study which used two measures of cognition taken 6 years apart (Mol et al. 2006), and found no relationship between baseline SMC and subsequent cognitive decline.

The focus of our analyses was the consistency of SMC reported in two initial waves (4 years apart). Other than consistency, we note that further complexities around the perception of SMC should also be considered in future studies. For example, while SMC without worry was independently associated with an increased risk of developing dementia compared to those without SMC, SMC with worry roughly doubled that risk (Jessen 2010). Although anxiety and depressive symptoms should always be considered as a confounder, our results suggest that the association between SMC and cognitive deficit or dementia cannot be wholly explained by these symptoms (Jessen et al. 2007; Jonker et al. 2000), evidenced by similar findings in those who had no anxiety or depression symptoms at baseline.

Our analyses add to the growing literature on the relationship between SMC and objective measures of cognitive function. When using a neuropsychological battery in the current study, the small change between waves in raw scores or time meant stable performance in our sample. Despite the 4-year intervals between waves, potential practice effects may still exist. Consistently, the slopes in the linear mixed models were mostly less than 0.2 SD or 3%, meaning that the differences in decline between SMC groups might be too subtle to be attested. Further work is required to confirm the utility of SMC as a robust indicator of future decline. Nevertheless, for people in their 60 s, SMC may be a useful indicator of an acute or contemporaneous drop in cognitive function.

Strengths and limitations. The population: The current study used data from a large population-based sample broadly representative of the Australian population (Anstey et al. 2012) with participants from a narrow age range and 12 years of follow-up and the opportunity to evaluate “sustained” and “remitting” SMC in addition to the more usual single time point assessment. Nevertheless, the selection of a population sample aged just 60–64, requirement for participation in follow-up visits, relatively short follow-up in the context of cognitive decline (3 waves over 8 years), may mean we were unable to detect patterns that may be visible in higher risk sections of the population or over longer time periods. It may also be that cognitive concerns expressed in early late-life are less likely to reflect actual, measurable decline than in a later life population (Jonker et al. 2000). Finally it may also be that attrition meant we missed the participants for whom this was most relevant, although our IPW analyses would suggest that this was not the case.

The assessment: The lack of structural and functional imaging and blood or cerebrospinal fluid based biomarkers for all participants to allow a biological contextualisation of our results inevitably limit our findings, as does use of a limited tool to assess SMC rather than a more complex assessment of SCD including nonmemory domains (Jessen et al. 2014; Molinuevo et al. 2017; Rabin et al. 2015; Smart et al. 2014). SMC may be sensitive but not specific in an early-late life population such as ours (Jessen 2010). Furthermore, the questions used to assess SMC in this study may have been open to varied interpretation by the participants. Specifically, participants might have interpreted the instruction to consider a time "earlier in life" as their teenage or early adulthood years or may have used a shorter time frame (e.g. 5 years ago). This may influence our results as those who answered “yes” to the first question did not proceed to the next two questions. Further, we categorized those who answered “don`t know” to the question on whether memory problems interfere with day-to-day life as experiencing SMC. Though there is no evidence against this approach, only a non-medical study in 1990s (Gilljam and Granberg 1993) supported it. It should be noted that 60 participants indicated “don`t know” at wave 1 and/or wave 2, including seven also reported that they had seen doctor about their memory.

## Conclusion

Despite the fact that people with SMC in their 60 s had poorer contemporaneous memory scores and impaired processing speed, there was no strong association between SMC, sustained or otherwise, and faster decline over follow up. Overall this implies that people may be able to perceive poorer performance but, whilst this may represent a decline from expected performance levels it does not necessarily indicate an at risk population for future decline. Further work is required to evaluate the fluctuating nature of SMC and its relationship with cognitive trajectories over longer periods of the life-course.

## Supplementary Information

Below is the link to the electronic supplementary material.

Supplementary file1 (DOCX 101 kb)

## Data Availability

The de-identified participant data that support the findings of this study are not publicly available but are available from the study committee upon reasonable request.

## Code availability

SAS code used for the analyses can be requested from the corresponding author.
