# Supplementary material for: No clear associations between subjective memory concerns and subsequent change in cognitive function: the PATH through life study
Source: Eur J Ageing. 2022 Mar 28;19(4):1181–8. doi: 10.1007/s10433-022-00694-2 (PMC9729657; doi:10.1007/s10433-022-00694-2)
Supplement: Supplementary file 1 — Supplementary file1 (DOCX 101 kb) [file 10433_2022_694_MOESM1_ESM.docx]

This supplementary has been provided by the authors to give readers additional information about their work.

Supplement to: Xu Y, Warwick J, Eramudugolla R, et al. No clear associations between subjective memory concerns and subsequent change in cognitive function: the PATH through life study


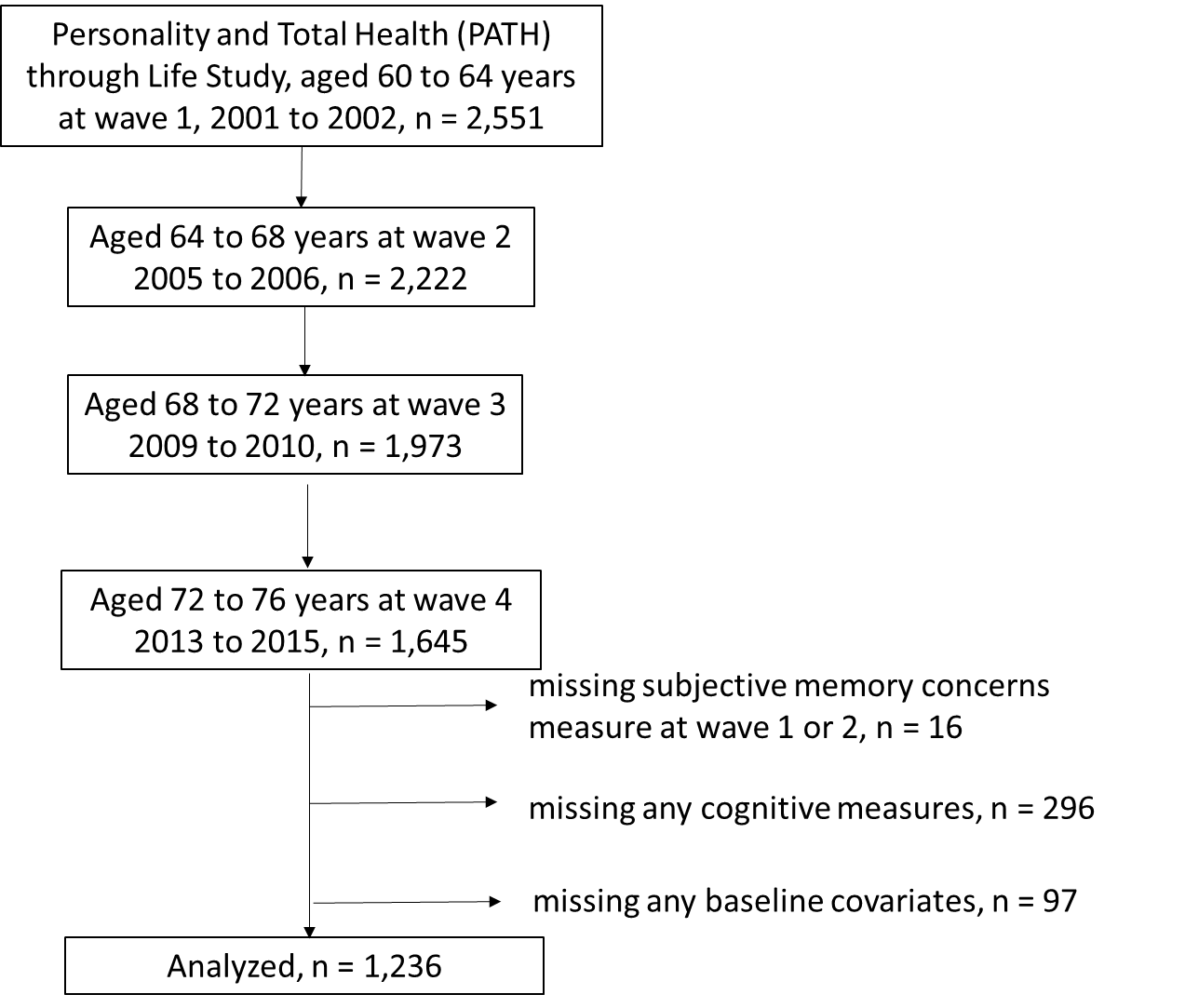


# Figure S1 Flow chart of participants included in the current analyses

# Table S1 Baseline characteristics of the participants included in the Personality and Total Health (PATH) through Life Study, and by those who were available at wave 4 and those who were lost to follow-up

| **Baseline characteristics** | The oldest of the three cohorts in the Personality and Total Health (PATH) through Life Study at wave 1  n = 2551 | |  | Breakdown of the oldest cohort (n = 2551) at wave 4 | | | | | | |
| --- | --- | --- | --- | --- | --- | --- | --- | --- | --- | --- |
|  |  |  |  | Lost to follow-up  n = 906 | |  | Followed up (n = 1645) | | | |
|  |  |  |  |  |  |  | Excluded | |  | Study sample |
|  |  |  |  |  |  |  | n = 409 | |  | n = 1236 |
| **Demographic at wave 1** |  | n |  |  | n |  |  | n |  |  |
| Female, n (%) | 1234 (48.4) | 2550 |  | 444 (49) | 906 |  | 210 (51.5) | 408 |  | 580 (46.9) |
| Age, years, mean ± SD | 62 (61, 64) | 2549 |  | 62 (61, 64) | 905 |  | 62.4 ± 1.5 | 408 |  | 62.5 ± 1.5 |
| Education, years, mean ± SD | 13.8 ± 2.8 | 2423 |  | 13.1 ± 3 | 869 |  | 13.8 ± 3 | 318 |  | 14.3 ± 2.6 |
| Caucasian (versus Asian and other), n (%) | 2441 (95.8) | 2548 |  | 851 (94) | 905 |  | 386 (94.8) | 407 |  | 1204 (97.4) |
| English-speaking, n (%) | 2226 (87.4) | 2548 |  | 747 (82.5) | 905 |  | 348 (85.5) | 407 |  | 1131 (91.5) |
| **Dementia risk factors at wave 1** |  |  |  |  |  |  |  |  |  |  |
| Body mass index, kilogram/meters^2^, median (IQR) | 26.2 (23.7, 28.9) | 2550 |  | 26.2 (23.8, 29.1) | 905 |  | 26.3 (23.5, 29.2) | 409 |  | 26.1 (23.8, 28.8) |
| Diabetes (self-reported), n (%) | 193 (7.6) | 2547 |  | 88 (9.7) | 904 |  | 30 (7.4) | 407 |  | 75 (6.1) |
| Raised blood pressure, systolic >140 mmHg or diastolic >90 mmHg, on or off antihypertensive medication, n (%) | 1277 (51.4) | 2486 |  | 462 (52.7) | 876 |  | 187 (50) | 374 |  | 628 (50.8) |
| Systolic blood pressure, mmHg, mean ± SD | 139.8 ± 19.5 | 2486 |  | 140 ± 20.2 | 876 |  | 139.1 ± 19.7 | 374 |  | 139.3 ± 18.9 |
| Diastolic blood pressure, mmHg, mean ± SD | 83 ± 10.7 | 2486 |  | 83 ± 11 | 876 |  | 82.9 ± 11.4 | 374 |  | 82.9 ± 10.3 |
| Traumatic brain injury (self-reported), n (%) | 142 (5.6) | 2547 |  | 62 (6.9) | 904 |  | 25 (6.1) | 407 |  | 55 (4.5) |
| Score of > 2 on Goldberg depression scale, n (%) | 650 (25.6) | 2539 |  | 294 (32.7) | 899 |  | 112 (27.7) | 404 |  | 244 (19.7) |
| Score of > 5 on Goldberg anxiety scale, n (%) | 288 (11.3) | 2539 |  | 127 (14.1) | 899 |  | 47 (11.6) | 404 |  | 114 (9.2) |
| Smoke (past or current versus never), n (%) | 1227 (48.2) | 2547 |  | 495 (54.8) | 904 |  | 194 (47.7) | 407 |  | 538 (43.5) |
| Physical activities |  |  |  |  |  |  |  |  |  |  |
| Mild (less frequent than “three times a week or more”), n (%) | 784 (30.9) | 2538 |  | 325 (36.2) | 898 |  | 122 (30.2) | 404 |  | 337 (27.3) |
| Moderate (less frequent than “once or twice a week”), n (%) | 868 (34.2) | 2538 |  | 358 (39.9) | 898 |  | 151 (37.4) | 404 |  | 359 (29.1) |
| Vigorous (less frequent than “one to three times a month”), n (%) | 1573 (62) | 2538 |  | 614 (68.4) | 898 |  | 255 (63.1) | 404 |  | 704 (57) |
| **Cognitive performance at wave 2** |  |  |  |  |  |  |  |  |  |  |
| Mini-Mental State Examination, median (IQR) | 30 (29, 30) | 2168 |  | 29 (28, 30) | 563 |  | 29.5 (29, 30) | 372 |  | 30 (29, 30) |
| Range | 10 to 147 |  |  | 14 to 147 |  |  | 21 to 30 |  |  | 21 to 30 |
| California Verbal Learning Test immediate recall, mean ± SD | 6.9 ± 2.2 | 2182 |  | 6.6 ± 2.3 | 569 |  | 6.6 ± 2.2 | 377 |  | 7.2 ± 2.2 |
| Range | 0 to 16 |  |  | 1 to 15 |  |  | 0 to 12 |  |  | 1 to 16 |
| California Verbal Learning Test delayed recall, mean ± SD | 6.1 ± 2.4 | 2182 |  | 5.8 ± 2.5 | 569 |  | 5.8 ± 2.3 | 377 |  | 6.3 ± 2.3 |
| Range | 0 to 16 |  |  | 0 to 16 |  |  | 0 to 11 |  |  | 0 to 16 |
| Symbol Digit Modalities, mean ± SD | 49.3 ± 9.4 | 2178 |  | 46.5 ± 9.8 | 569 |  | 48.3 ± 9.5 | 373 |  | 50.9 ± 8.8 |
| Range | 0 to 10 |  |  | 0 to 10 |  |  | 15 to 74 |  |  | 5 to 80 |
| Trail making test part A time, seconds, median (IQR) | 33 (27, 40) | 2183 |  | 35 (28, 44) | 571 |  | 33 (28, 39.5) | 376 |  | 32 (27, 39) |
| Range | 10 to 300 |  |  | 10 to 300 |  |  | 10 to 110 |  |  | 13 to 100 |
| Trail making test part B time, seconds, median (IQR) | 74 (59, 93) | 2163 |  | 81 (66, 107) | 558 |  | 74 (59, 96) | 369 |  | 71 (58, 88) |
| Range | 19 to 30 |  |  | 19 to 30 |  |  | 12 to 300 |  |  | 29 to 300 |
| Digit span backwards, mean ± SD | 5.1 ± 2.2 | 2159 |  | 4.6 ± 2.1 | 560 |  | 5 ± 2.3 | 363 |  | 5.4 ± 2.2 |
| Range | 4 to 80 |  |  | 4 to 76 |  |  | 1 to 10 |  |  | 0 to 10 |
| Purdue pegboard dominant hand, mean ± SD | 13.4 ± 2.1 | 2174 |  | 13.1 ± 2.3 | 565 |  | 13.4 ± 2.1 | 373 |  | 13.6 ± 2 |
| Range | 3 to 25 |  |  | 5 to 20 |  |  | 5 to 25 |  |  | 3 to 21 |
| Purdue pegboard non-dominant hand, mean ± SD | 12.7 ± 2 | 2161 |  | 12.3 ± 2.2 | 561 |  | 12.6 ± 2 | 364 |  | 12.9 ± 1.8 |
| Range | 4 to 19 |  |  | 5 to 19 |  |  | 4 to 17 |  |  | 6 to 18 |
| Purdue pegboard both hands, mean ± SD | 10.4 ± 1.8 | 2159 |  | 10 ± 2 | 561 |  | 10.4 ± 1.9 | 362 |  | 10.6 ± 1.7 |
| Range | 2 to 19 |  |  | 2 to 19 |  |  | 3 to 15 |  |  | 3 to 15 |

# Table S2 Covariates in the linear mixed model evaluating the relationship between subjective memory concerns and objectively measured cognition

|  | CVLT immediate recall | CVLT delayed recall | Symbol Digit Modalities | Trail making test part A time | Trail making test part B time | Digit span backwards | Purdue pegboard (dominant) | Purdue pegboard (non-dominant) | Purdue pegboard (both) |
| --- | --- | --- | --- | --- | --- | --- | --- | --- | --- |
| Age (per year increase) | **-0.04 (-0.07, -0.01), 0.005** | -0.03 (-0.06, 0), 0.07 | **-0.04 (-0.07, 0), 0.03** | **1.01 (1.01, 1.02), 0.0009** | **1.01 (1, 1.03), 0.01** | **-0.03 (-0.07, 0), 0.02** | **-0.05 (-0.08, -0.02), 0.001** | **-0.05 (-0.08, -0.02), 0.002** | **-0.04 (-0.07, 0), 0.03** |
| Male vs female | **-0.49 (-0.58, -0.4), <0.0001** | **-0.53 (-0.63, -0.43), <0.0001** | **-0.19 (-0.29, -0.08), 0.0006** | 1.02 (0.99, 1.05), 0.18 | 1.02 (0.98, 1.05), 0.39 | 0.07 (-0.03, 0.16), 0.19 | **-0.56 (-0.65, -0.46), <0.0001** | **-0.28 (-0.38, -0.18), <0.0001** | **-0.38 (-0.48, -0.28), <0.0001** |
| English speaking (yes vs. no) | **0.34 (0.19, 0.49), <0.0001** | **0.3 (0.13, 0.47), 0.0005** | **0.44 (0.27, 0.62), <0.0001** | **0.86 (0.82, 0.9), <0.0001** | **0.8 (0.75, 0.85), <0.0001** | **0.48 (0.32, 0.65), <0.0001** | -0.13 (-0.29, 0.03), 0.11 | -0.09 (-0.25, 0.08), 0.31 | -0.1 (-0.27, 0.07), 0.24 |
| Years in education  (per year increase) | **0.08 (0.06, 0.1), <0.0001** | **0.07 (0.05, 0.08), <0.0001** | **0.09 (0.08, 0.11), <0.0001** | **0.99 (0.99, 1), 0.0004** | **0.97 (0.97, 0.98), <0.0001** | **0.09 (0.07, 0.11), <0.0001** | **0.03 (0.01, 0.05), 0.0004** | **0.04 (0.02, 0.06), <0.0001** | **0.04 (0.02, 0.06), <0.0001** |
| Symptoms of anxiety and/or depression (yes vs. no) | -0.04 (-0.14, 0.06), 0.43 | -0.06 (-0.18, 0.06), 0.32 | **-0.13 (-0.25, 0), 0.05** | 1.01 (0.98, 1.04), 0.62 | 1.03 (0.99, 1.08), 0.14 | -0.01 (-0.13, 0.1), 0.82 | -0.08 (-0.19, 0.03), 0.16 | -0.02 (-0.14, 0.09), 0.7 | -0.12 (-0.23, 0), 0.05 |
| Body mass index  (per unit increase in kg/m^2^) | 0 (-0.01, 0.01), 0.82 | -0.01 (-0.02, 0.01), 0.35 | -0.01 (-0.02, 0.01), 0.36 | 1 (1, 1), 0.84 | 1 (1, 1), 0.82 | 0 (-0.01, 0.01), 0.93 | **-0.03 (-0.04, -0.02), <0.0001** | **-0.03 (-0.04, -0.02), <0.0001** | **-0.03 (-0.04, -0.01), <0.0001** |
| Self-reported diabetes  (yes vs. no) | -0.05 (-0.23, 0.12), 0.55 | -0.15 (-0.35, 0.05), 0.13 | -0.17 (-0.38, 0.04), 0.11 | **1.07 (1.01, 1.13), 0.02** | 1.05 (0.98, 1.12), 0.21 | -0.14 (-0.33, 0.06), 0.16 | **-0.29 (-0.48, -0.1), 0.003** | **-0.47 (-0.67, -0.27), <0.0001** | -0.39 (-0.59, -0.19), 0.0002 |
| Hypertension  (yes vs. no) | 0.02 (-0.06, 0.1), 0.62 | 0.03 (-0.06, 0.13), 0.51 | -0.04 (-0.14, 0.06), 0.47 | 1 (0.97, 1.03), 1 | 0.99 (0.96, 1.03), 0.77 | 0.01 (-0.08, 0.11), 0.76 | -0.08 (-0.17, 0.01), 0.1 | -0.08 (-0.18, 0.01), 0.08 | -0.07 (-0.17, 0.02), 0.14 |
| Ever smoking vs. never | 0 (-0.08, 0.09), 0.92 | 0.03 (-0.07, 0.13), 0.55 | 0.07 (-0.04, 0.17), 0.21 | 1 (0.97, 1.03), 1 | 0.99 (0.95, 1.02), 0.42 | 0.06 (-0.03, 0.15), 0.21 | -0.06 (-0.15, 0.03), 0.21 | -0.01 (-0.1, 0.09), 0.86 | -0.01 (-0.11, 0.08), 0.78 |
| Traumatic brain injury (yes vs. no) | -0.01 (-0.21, 0.18), 0.91 | -0.01 (-0.23, 0.22), 0.96 | 0.05 (-0.19, 0.29), 0.7 | 1 (0.94, 1.07), 0.94 | 0.98 (0.91, 1.06), 0.64 | 0 (-0.22, 0.22), 0.98 | -0.07 (-0.28, 0.14), 0.51 | -0.13 (-0.36, 0.09), 0.24 | -0.18 (-0.41, 0.04), 0.11 |
| Mild physical activity less frequent than three times a week (yes vs. no) | **-0.1 (-0.2, -0.01), 0.04** | **-0.14 (-0.26, -0.03), 0.01** | -0.01 (-0.13, 0.11), 0.89 | 1.02 (0.99, 1.05), 0.29 | **1.04 (1, 1.09), 0.03** | -0.03 (-0.14, 0.08), 0.59 | 0.04 (-0.06, 0.15), 0.43 | 0.01 (-0.1, 0.12), 0.86 | 0.02 (-0.1, 0.13), 0.77 |
| Moderate physical activity less frequent than once a month (yes vs. no) | -0.03 (-0.13, 0.07), 0.54 | 0.02 (-0.09, 0.14), 0.71 | 0.03 (-0.09, 0.15), 0.63 | 1.01 (0.98, 1.04), 0.49 | 1 (0.96, 1.04), 1 | 0 (-0.11, 0.12), 0.93 | 0.06 (-0.05, 0.16), 0.31 | -0.11 (-0.22, 0), 0.06 | -0.01 (-0.13, 0.1), 0.8 |
| Rigorous physical activity less frequent than once a month (yes vs. no) | -0.01 (-0.1, 0.08), 0.78 | 0.05 (-0.05, 0.15), 0.3 | 0.07 (-0.04, 0.17), 0.22 | 1.01 (0.99, 1.04), 0.34 | 0.99 (0.95, 1.02), 0.42 | 0.05 (-0.04, 0.15), 0.28 | -0.03 (-0.12, 0.07), 0.55 | 0.07 (-0.03, 0.17), 0.17 | 0.02 (-0.08, 0.13), 0.65 |

CVLT denotes California Verbal Learning Test.

All co-variates are from the baseline assessment. Numbers are estimations with 95% confidence intervals with p values, indicating the differences in cognitive performance at wave 2 by covariate status. Time (seconds) used for trail making tests part A and B was log-transformed to form normal distributions. For ease of interpretation, we exponentiated the coefficients for trail making tests part A and B, e.g. an exponentiated coefficient of 1.01 represents a 1% increase in time.

# Table S3 Associations between subjective memory concerns and cognition in the linear mixed model^a^

|  | **Inverse probability weighting (n = 1236)** | | | |  | **Among those who did not have anxiety or depression symptoms at baseline (n = 957)** | | | |
| --- | --- | --- | --- | --- | --- | --- | --- | --- | --- |
|  | Cognitive performance at wave 2 (column a) | | Cognitive change between waves over the subsequent 8 years (column b) | |  | Cognitive performance at wave 2 (column a) | | Cognitive change between waves over the subsequent 8 years (column b) | |
|  |  |  |  |  |  |  |  |  |  |
|  | β_2_^b^ (95% CI) | P value | β_3_^b^ (95% CI) | P value |  | β_2_^b^ (95% CI) | P value | β_3_^b^ (95% CI) | P value |
| CVLT immediate recall |  | **0.02** |  | 0.21 |  |  | 0.09 |  | 0.09 |
| “remitting” vs “no” | **-0.22 (-0.42, -0.02)** | **0.03** | 0.02 (-0.09, 0.13) | 0.69 |  | -0.26 (-0.54, 0.01) | 0.06 | 0.08 (-0.07, 0.24) | 0.28 |
| “new-onset” vs “no” | **-0.25 (-0.45, -0.05)** | **0.01** | **0.12 (0.01, 0.23)** | **0.04** |  | -0.24 (-0.51, 0.03) | 0.08 | **0.18 (0.03, 0.33)** | **0.02** |
| “sustained” vs “no” | -0.11 (-0.33, 0.1) | 0.30 | -0.01 (-0.13, 0.1) | 0.82 |  | -0.12 (-0.42, 0.18) | 0.43 | -0.01 (-0.17, 0.16) | 0.92 |
| CVLT delayed recall |  | 0.35 |  | 0.20 |  |  | 0.49 |  | 0.31 |
| “remitting” vs “no” | -0.13 (-0.35, 0.09) | 0.24 | -0.05 (-0.25, 0.15) | 0.63 |  | -0.13 (-0.43, 0.17) | 0.39 | 0.01 (-0.27, 0.28) | 0.96 |
| “new-onset” vs “no” | -0.15 (-0.36, 0.07) | 0.18 | 0.11 (-0.09, 0.31) | 0.28 |  | -0.19 (-0.48, 0.09) | 0.18 | 0.11 (-0.15, 0.38) | 0.41 |
| “sustained” vs “no” | -0.1 (-0.32, 0.13) | 0.42 | -0.18 (-0.39, 0.02) | 0.08 |  | -0.03 (-0.35, 0.28) | 0.83 | -0.25 (-0.54, 0.05) | 0.10 |
| Symbol Digit Modalities |  | 0.12 |  | 0.18 |  |  | 0.20 |  | 0.60 |
| “remitting” vs “no” | -0.14 (-0.36, 0.09) | 0.23 | -0.08 (-0.16, 0) | 0.05 |  | -0.1 (-0.4, 0.2) | 0.52 | -0.08 (-0.19, 0.04) | 0.18 |
| “new-onset” vs “no” | -0.06 (-0.28, 0.16) | 0.59 | 0.01 (-0.07, 0.09) | 0.80 |  | -0.11 (-0.4, 0.18) | 0.46 | 0 (-0.11, 0.11) | 1.00 |
| “sustained” vs “no” | **-0.26 (-0.5, -0.03)** | **0.03** | 0.04 (-0.05, 0.12) | 0.37 |  | **-0.33 (-0.65, -0.01)*** | **0.05*** | 0.02 (-0.1, 0.14) | 0.80 |
| Trails A |  | **0.04** |  | 0.23 |  |  | **0.01*** |  | **0.04*** |
| “remitting” vs “no” | **1.08 (1.01, 1.15)** | **0.02** | 0.99 (0.96, 1.02) | 0.52 |  | 1.09 (1, 1.18) | 0.06 | 0.99 (0.95, 1.04) | 0.82 |
| “new-onset” vs “no” | 1.07 (1, 1.14) | 0.05 | 0.97 (0.94, 1) | 0.07 |  | **1.12 (1.04, 1.22)*** | **0.006*** | **0.94 (0.9, 0.98)*** | **0.004*** |
| “sustained” vs “no” | 1 (0.93, 1.06) | 0.89 | 1.01 (0.98, 1.05) | 0.44 |  | 1.01 (0.93, 1.11) | 0.76 | 1.01 (0.96, 1.05) | 0.83 |
| Trails B |  | 0.67 |  | 0.81 |  |  | 0.36 |  | 0.29 |
| “remitting” vs “no” | 1.01 (0.93, 1.09) | 0.84 | 1.01 (0.97, 1.04) | 0.77 |  | 1.09 (0.98, 1.21) | 0.10 | 1.01 (0.97, 1.07) | 0.56 |
| “new-onset” vs “no” | 1.03 (0.95, 1.11) | 0.46 | 1 (0.97, 1.04) | 0.82 |  | 1.02 (0.92, 1.13) | 0.70 | 1.03 (0.98, 1.08) | 0.19 |
| “sustained” vs “no” | 1.05 (0.96, 1.14) | 0.29 | 0.98 (0.95, 1.02) | 0.38 |  | 1.04 (0.93, 1.16) | 0.49 | 0.97 (0.92, 1.02) | 0.22 |
| Digit span backwards |  | **0.04** |  | 0.33 |  |  | 0.38 |  | 0.46 |
| “remitting” vs “no” | 0.2 (-0.01, 0.42) | 0.06 | -0.05 (-0.14, 0.05) | 0.34 |  | 0 (-0.29, 0.28) | 0.98 | -0.02 (-0.15, 0.11) | 0.77 |
| “new-onset” vs “no” | -0.12 (-0.33, 0.09) | 0.27 | 0.07 (-0.02, 0.17) | 0.13 |  | -0.04 (-0.31, 0.24) | 0.80 | 0.06 (-0.06, 0.19) | 0.33 |
| “sustained” vs “no” | -0.19 (-0.41, 0.04) | 0.10 | 0.01 (-0.09, 0.11) | 0.88 |  | -0.27 (-0.58, 0.03) | 0.08 | 0.09 (-0.05, 0.23) | 0.21 |
| Purdue pegboard (dominant) |  | 0.28 |  | 0.64 |  |  | 0.16 |  | 0.84 |
| “remitting” vs “no” | -0.18 (-0.39, 0.04) | 0.11 | 0.04 (-0.07, 0.16) | 0.47 |  | -0.26 (-0.55, 0.03) | 0.08 | 0 (-0.17, 0.16) | 0.96 |
| “new-onset” vs “no” | -0.11 (-0.33, 0.1) | 0.30 | -0.06 (-0.17, 0.05) | 0.30 |  | -0.19 (-0.47, 0.09) | 0.18 | -0.07 (-0.23, 0.08) | 0.37 |
| “sustained” vs “no” | -0.11 (-0.34, 0.12) | 0.34 | -0.01 (-0.12, 0.11) | 0.93 |  | -0.13 (-0.44, 0.17) | 0.39 | -0.02 (-0.19, 0.15) | 0.84 |
| Purdue pegboard (non-dominant) |  | **0.006** |  | 0.10 |  |  | **0.05*** |  | **0.04*** |
| “remitting” vs “no” | **-0.27 (-0.5, -0.04)** | **0.02** | -0.01 (-0.14, 0.11) | 0.83 |  | **-0.33 (-0.64, -0.02)*** | **0.04*** | -0.01 (-0.19, 0.16) | 0.87 |
| “new-onset” vs “no” | 0.01 (-0.22, 0.24) | 0.93 | **-0.15 (-0.27, -0.02)** | **0.02** |  | 0.09 (-0.22, 0.39) | 0.58 | **-0.24 (-0.41, -0.08)*** | **0.004*** |
| “sustained” vs “no” | **-0.35 (-0.59, -0.11)** | **0.005** | 0.05 (-0.08, 0.17) | 0.49 |  | -0.32 (-0.65, 0.01) | 0.06 | 0.04 (-0.15, 0.22) | 0.69 |
| Purdue pegboard (both) |  | 0.59 |  | 0.67 |  |  | 0.22 |  | 0.55 |
| “remitting” vs “no” | -0.1 (-0.32, 0.13) | 0.41 | -0.05 (-0.16, 0.07) | 0.41 |  | -0.2 (-0.5, 0.1) | 0.19 | -0.04 (-0.19, 0.12) | 0.63 |
| “new-onset” vs “no” | -0.12 (-0.35, 0.1) | 0.28 | 0.02 (-0.09, 0.14) | 0.69 |  | -0.25 (-0.54, 0.04) | 0.09 | 0.06 (-0.09, 0.21) | 0.45 |
| “sustained” vs “no” | -0.08 (-0.32, 0.16) | 0.53 | -0.05 (-0.17, 0.07) | 0.41 |  | 0 (-0.32, 0.32) | 1.00 | -0.09 (-0.26, 0.07) | 0.27 |

CVLT denotes California Verbal Learning Test, CI confidence interval.

^a^CVLT immediate recall, Symbol Digit Modalities, Trails A and B, digit span backwards and Purdue pegboard wave 2 to wave 4; CVLT delayed recall wave 2 to wave 3. For CVLT delayed recall, it is the subsequent 4 years, rather than 8 years.

^b^Cognitive test scores = intercept + β_1_ X time (i.e. wave 2 coded as 0, wave 3 coded as 1 and wave 4 coded as 2) + β_2_ X subjective memory concerns category (0=”no”, 1=”remitting”, 2=”new-onset”, 3=”sustained”) + β_3_ X time X subjective memory concerns category + adjusted variables

Adjusted for age, sex, English speaking, education, symptoms of anxiety and/or depression, body mass index, self-reported diabetes, hypertension, traumatic brain injury, smoking and physical activity.

Time (seconds) used for trail making tests part A and B was log-transformed to form normal distributions. For ease of interpretation, we exponentiated the coefficients for trail making tests part A and B, e.g. an exponentiated coefficient of 1.01 represents a 1% increase in time.
